# Modification of bacterial cells for *in vivo* remotely guided systems

**DOI:** 10.3389/fbioe.2022.1070851

**Published:** 2023-01-04

**Authors:** Iaroslav Rybkin, Sergey Pinyaev, Olga Sindeeva, Sergey German, Maja Koblar, Nikolay Pyataev, Miran Čeh, Dmitry Gorin, Gleb Sukhorukov, Aleš Lapanje

**Affiliations:** ^1^ Helmholtz-Zentrum Dresden-Rossendorf, Leipzig, Germany; ^2^ Jožef Stefan Institute, Ljubljana, Slovenia; ^3^ State University, Saratov, Russia; ^4^ Jožef Stefan International Postgraduate School, Ljubljana, Slovenia; ^5^ National Research Ogerev Mordovia State University, Saransk, Russia; ^6^ A.V. Zelman Center for Neurobiology and Brain Rehabilitation, Skolkovo Institute of Science and Technology, Moscow, Russia; ^7^ Center of Photonic Science and Engineering, Skolkovo Institute of Science and Technology, Moscow, Russia; ^8^ Institute of Spectroscopy of the Russian Academy of Sciences, Moscow, Russia; ^9^ Queen Mary University of London, London, United Kingdom

**Keywords:** bacterial therapy, guiding of cells, encapsulation, cancer treatment, electrostatic interaction, modification of cell surface, layer-by-layer approach, magnetic field

## Abstract

It was shown recently that bacterial strains, which can act specifically against malignant cells, can be used efficiently in cancer therapy. Many appropriate bacterial strains are either pathogenic or invasive and there is a substantial shortage of methods with which to monitor *in vivo* the distribution of bacteria used in this way. Here, it is proposed to use a Layer-by-Layer (LbL) approach that can encapsulate individual bacterial cells with fluorescently labeled polyelectrolytes (PE)s and magnetite nanoparticles (NP)s. The NP enable remote direction *in vivo* to the site in question and the labeled shells in the far-red emission spectra allow non-invasive monitoring of the distribution of bacteria in the body. The magnetic entrapment of the modified bacteria causes the local concentration of the bacteria to increase by a factor of at least 5. The PEs create a strong barrier, and it has been shown in *vitro* experiments that the division time of bacterial cells coated in this way can be regulated, resulting in control of their invasion into tissues. That animals used in the study survived and did not suffer septic shock, which can be attributed to PE capsules that prevent release of endotoxins from bacterial cells.

## 1 Introduction

Using a top-down engineering approach, an ideal cancer therapy that can be envisioned involves micro- or nanosized programmable “robot devices” that: (i) are self-propelled and specifically target tumors preferentially, (ii) are selectively cytotoxic only to cancer cells and (iii) respond to external signals, which can transfer information about the local environment and enable remote guidance (Forbes, 2010). In order to specifically target tumors the agent should accumulate in the tumor either due to the intrinsic properties of tumor tissues enabling EPR (Enhanced Permeability and Retention) effect (Fang et al., 2016) or by the property of the agent such as self-propulsion through the blood and penetration into tumor regions that cannot be accessed by passive systemic deliverable therapies. According to aforementioned criteria for ideal anticancer treatment, bacterial cells share most of these characteristics and in fact, there are genera, such as *Salmonella* (Pawelek et al., 1997), *Escherichia* (Yong et al., 2004), *Clostridium* (Parker et al., 1947; Malmgren and Flanigan, 1955), *Bifidobacterium* (Kohwi et al., 1978), *Caulobacter* (Bhatnagar et al., 2006), *Listeria* (Pan et al., 1999; Kim et al., 2009), *Proteus* (Arakawa et al., 1968), and *Streptococcus* (Maletzki et al., 2008), which have been shown in laboratory experiments *in vitro* (reviewed by Duong et al., 2019; Badie et al., 2021; Yang et al., 2021), and in Phase 1 clinical *trials in vivo* (reviewed by Gupta et al., 2021) to be found preferentially within tumors and lysed cancerous cells.

Although selected bacteria in most cases can be efficiently used in laboratories, their clinical use is currently limited by the lack of strategies for: (*i*) efficient delivery of bacteria to the desired location, (*ii*) protection of the host from direct invasion by bacteria or hyperactivity of the immune system triggered, for example, by endotoxins and (*iii*) remote monitoring of distribution of applied bacterial cells *in vivo*. According to Forbes et al. (2003) bacteria used in therapy are not directed specifically to the tumor in question, and their accumulation within a tumor is a passive process (Forbes, 2010). Normally, when bacteria enter the circulatory system, they are distributed evenly throughout the organs and tissues in the body. Only the local conditions in tumors, where the activity of the immune system is minimized, enable the survival and propagation of bacteria (Forbes et al., 2003). Consequently, the specificity of the treatment is dependent on the type of bacterial cells, which may or may not multiply in tumor tissue, depending on the responsiveness of the host immune system, the local blood supply and other factors. Determining these factors prior to treatment and monitoring them during the procedure are difficult or impossible, but necessary to achieve the most efficient and safe treatment. Bacteria and their cellular components are extremely strong immune stimulators with endo- and exotoxins whose systemic delivery increases the risk of sepsis as well as development of toxic syndrome (Schletter et al., 1995), since the only available control of their propagation in such approaches being with antibiotics. Due to these obstacles, the bacterial therapy on the one hand cannot be easily applied to patients and the current use of bacteria does not enable use of a wider repertoire of bacterial species although some, such as *Pseudomonas* strains might possess very potent toxins against cancer cells (Weldon and Pastan, 2011). With these drawbacks especially to increase the repertoire of bacteria that can be used by specific delivery to the tumors, and monitoring their distribution and controlling their growth, we propose the robust solution without using any genetic modifications of bacteria. Genetic manipulation, which enables introduction of specific receptors, toxins and genetic constructs that can control propagation (Bernardes et al., 2013; Piñero-Lambea et al., 2015; Riglar and Silver, 2018), is currently needed to be further improved for preparing bacteria for therapeutic applications. This is because we are limited to the genetic tools that are applied only to certain clinically relevant bacterial genera. They are time consuming, since the genetic constructs must be extensively tested, and the addition of a new trait to the particular strain can diminish or interfere with the needed physiological properties of unmodified bacteria e.g. virulence or specific genes involved in targeting (reviewed by Duong et al., 2019). As discussed by Forbes et al., 2018), it has become clear even with the limited clinical studies that substantial bacterial colonization of tumors is required for any significant clinical benefit. Properties such as toxicity, cancer tropism and genetic stability during the bacterial growth are also currently not the most potent among used bacteria (Forbes et al., 2018). Consequently, there is a substantial need to improve these properties and develop approaches that can be applied on the wide repertoire of bacterial species. One of such general property of bacterial cells is the surface electronegativity, which can help overcome aforementioned obstacles in improving bacterial cells used in bacterial-based therapies.

Since bacterial cells are negatively charged, they can act as a core for deposition of oppositely charged PE layers on their surfaces. Such deposition uses a LbL approach which forms a designed core-shell structure. Deposition of the PE on the cell surface can add new modalities to the bacteria such as improved adhesive properties and survivability in harsh conditions (Anselmo et al., 2016). It can also introduce new physiological activities such as increased specific binding to cells, or expand the spectrum of action by modification of PEs with fluorescent dyes, enzymes or ligands such as antibodies (Ai et al., 2003; Campas and O’Sullivan, 2003; Johnston et al., 2006; Costa and Mano, 2014), which can in principle allow cells to be monitored *in vivo.* For the remote targeting of bacteria, different sorts of nanoparticles can be incorporated within the layers, and could be used for the controlled delivery as has been shown in inanimate delivery systems using nano- and microparticles (Fakhrullin and Lvov, 2012). It is expected that such shells will separate the surface of the cell from the immune system and due to the shell strength, the division rate can be controlled by the capsules (Rybkin et al., 2019). This will allow control of the invasiveness of the delivered bacteria. Accordingly, our main aims in this study were to develop a proof of concept for *in vivo* guidance, detection and distribution of shell-coated bacteria within the mammalian body and to control the growth of the bacteria *in vitro.* The goal was to overcome the aforementioned obstacles in bacterial therapy, to make the bacterial therapies safer, and to broaden the spectra of the targets of different bacterial strains. Our specific aims were: (*i*) to develop a method for remote guidance of bacterial cells using a magnetic field, (*ii*) to perform a study of the feasibility of monitoring the distribution of bacterial cells inside the mammalian body by entrapping bacteria within fluorescently labeled capsules and (*iii*) to examine LbL encapsulation of bacteria as a method to control their population growth rate as one of the important pathogenic factors.

## 2 Materials and methods

### 2.1 Bacterial strains and growth conditions

Our experimental simulation of targeted bacterial therapy used cells of non-motile *Escherichia coli* TOP 10 strain (F–mcrA Δ(mrr-hsdRMS-mcrBC) Φ80lacZΔM15 ΔlacX74 recA1 araD139 Δ(ara leu) 7697 galU galK rpsL (StrR) endA1 nupG), containing pRSET-emGFP plasmid. The plasmid contains the T7 promoter region upstream of the emGFP reporter gene and ApR cassette added to the streptomycin intrinsic resistance, which enables observation of cells with a confocal microscope and control of bacterial contaminants by amending ampicillin in media. Cells were cultivated at 37 °C on nutrient agar plates (Sigma-Aldrich) supplemented with ampicillin (NAamp, 100 μg ml^−1^, Sigma-Aldrich). From the overnight culture, 1 ml was transferred into 100 ml of the fresh medium and incubated until obtaining OD_600_ = 0.2 (measured with reduced optical path of 200 µL well of a 96-well plate). All liquid cultures were incubated by shaking on a rotary shaker at 37°C and 150 rpm. Prior to observation, expression of emGFP was not induced since the low leakage expression was sufficiently intense for studies by confocal microscopy.

### 2.2 Preparation of PEs for layer-by-layer coating

Negatively charged sodium poly (styrene sulfonate) (PSS, MW = 70 kDa) and positively charged poly (ethyleneimine) PEs (PEI, MW = 600–1000 kDa), both purchased from Sigma-Aldrich, were used for cell coating which was based on the electrostatic principle (Sukhorukov et al., 1998). The solutions of PEs in Milli-Q (18.2 MΩ cm) water (2.5 mg ml^−1^, pH seven adjusted with NaOH or HCl) were prepared by dissolving PEs with stirring and then by sonication (35kHz, 100W, 15min). PEI was stained with Cyanine7 (Cy7, Lumiprobe, United States) according to the protocol of N-Hydroxysuccinimide (NHS) ester labeling of amino biomolecules (Lomant and Fairbanks, 1976). Cy7 solution (9 mg in 9 ml of DMSO) was added to PEI solution (2.5 mg ml^−1^ in 40 ml of water at pH 8.4) in a 50 ml tube and incubated for 4 h at room temperature—rt (20°C) under constant stirring. To remove the residual dye after labeling, the solution was dialyzed for 3 days against MQ water using a dialysis tube (Orange Scientific) with nominal molecular weight limits between 12 and 14 kDa and titrated up to pH seven at the end of the dialysis. All the PEs were sterilized by filtration through 0.2 µm sterile filters.

### 2.3 Coating procedure

**FIGURE 1:**
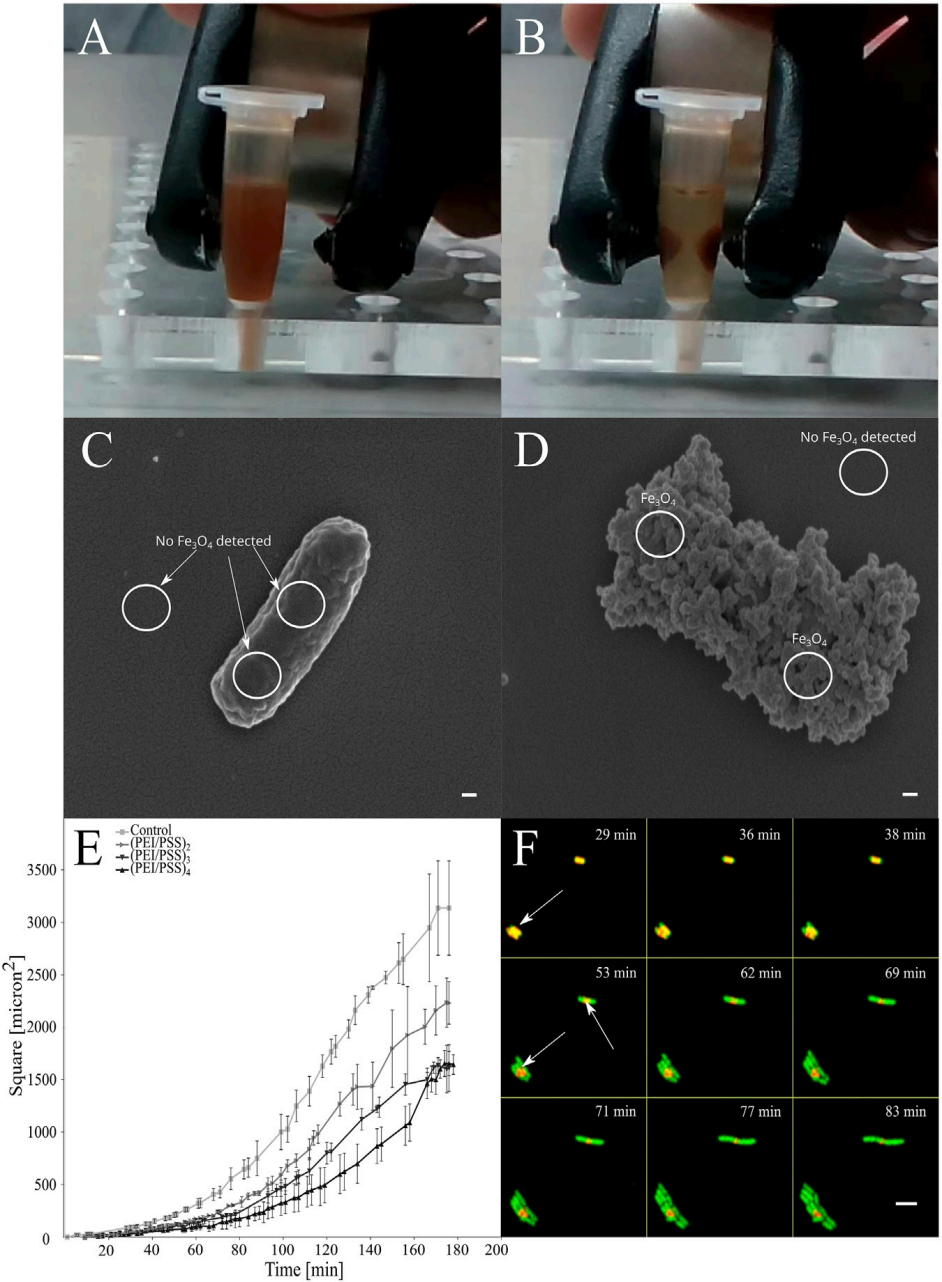
*In vitro* magnetic entrapment of the *E. coli* cells coated by PEI)/(PSS)/PEI/Fe3O4. PEI = polyethylenimine, PSS = poly (styrene)sulfonate. **(A,B)** When a magnetic field is applied, movement occurs instantly. **(C,D)** Presence or absence of nanoparticles was confirmed by SEM (EDS), where cells that are covered with the PE layers where magnetite nanoparticles are incorporated in between the layers are showing a rough surface and presence of Fe, scale bars = 100 nm. **(E)** Effects of entrapment of cells in layers of polyelectrolytes on growth of the population of the individually coated bacterial cells. Growth rate is showed as the function of the coverage of the surface by bacterial cells in time according to the analysis of micrographs obtained with TLCM (see methods). **(F)** Escape of the cells from the LbL layers when entrapped in polyelectrolyte layers. The LbL capsule is red, indicated with arrows, GFP producing cells are green and overlap regions are yellow. Scale bar = 5 µm.

Prior to coating cells in PE layers, bacterial cells were grown at 37°C by shaking at 150 rpm until reaching OD_600_ = 0.2 (in 200 µL of a 96 well plate). The obtained culture was further concentrated by centrifugation of 50 ml of bacterial culture at 5,000 g for 5 min and washing the precipitate 3 times in 30 ml of 0.9% NaCl and finally resuspending it in 10 ml of 0.9% NaCl. The cells were then coated with layers of PEI/PSS/PEI/magnetite-NPs/(PEI-Cy7/PSS)_2_. Firstly, a layer of positively charged PEI was deposited by adding 0.25% (w/v) solution of PEI in MQ water (adjusted to pH = 7 with 1 M HCl) to the washed and concentrated cells with the final OD_600_ = 1.2 in 1:1 v/v ratio with PEI and incubated at rt for 5 min. Unattached PEI was washed out from the suspension which was then centrifuged at 900 g for 2 min. The obtained pellet was washed twice with 1 ml of 0.9% NaCl without resuspending the pellet. The PEI coated cells were washed with 0.9% NaCl solution and then the second negatively charged PSS layer was deposited at pH 7 (adjusted with 0.1 M NaOH) using the same procedure as for PEI except the cells were centrifuged at 1500 g for 3 min. After addition of a third PEI layer, magnetite NPs were added. The produced magnetite NPs were previously characterized by German et al. (2013) (German et al., 2013). The average size of NPs was 12 nm, while zeta potential was −47 ± 6 mV (Mayorova et al., 2020). The magnetite NPs were incubated with the cells for 5 min (1 mg ml^−1^ particles, 1:1 v/v particles to cells). The excess magnetite particles were washed out by centrifugation at 1500 *g* for 3 min. The coated cells were collected by a magnet, additionally washed 2 times by pipetting and then resuspended in 0.9% NaCl solution. After applying magnetite, further layers of PEI-Cy 7/PSS were deposited in the manner described above. The fluorescent spectrum of the coatings was analyzed using a Synergy H1 microplate reader (BioTek, United States) with excitation at 730 nm and emission at 750–800 nm (see SM, Supplementary Figure S1). Finally, 8 layers of PEs were deposited on the bacterial cells, one of these being a paramagnetic layer. At the end coated cells were attracted using the permanent magnet that enabled purification of the fraction of cells that is responsive to the magnetic field (the majority) from the fraction of cells that might not be well coated (see Figure 1B). The zeta potential of the coated cells in PE layers was previously assessed by Rybkin et al. (Rybkin et al., 2019).

### 2.4 Scanning electron microscopy (SEM) and energy-dispersive X-ray spectroscopy (EDS) analysis

To obtain the best possible images with SEM, samples were prepared using one of three different protocols: (i) air drying at rt (20°C) without any fixatives, (ii) freeze drying, and (iii) chemical fixation with drying at 40°C. For normal drying the samples of the coated and control cells (intact, free of magnetic NPs and PEs) were suspended in 0.009% NaCl solution and placed on silicon wafers and dried at rt (20°C) for 72 h. For freeze drying, the cells were resuspended in 0.009% NaCl solution and pre-frozen in liquid nitrogen. The samples were then placed in a freeze dryer (Christ Gamma 1–16 LSC Plus) where they were left for 72 h at −50 °C. For chemical fixation, the cells were prepared by as described previously (Sretenovic et al., 2017). Briefly, the cells were fixed with 0.5% glutaraldehyde and 1% formaldehyde in a 0.1 M cocodylate buffer 0.1 M and transferred to a clean glass slide. After rewashing with cocodylate, 1% osmium tetroxide was added, and the sample was left for 1 h. Then the samples were rinsed with water and incubated for 5 min in ethanol of different dilutions (30%, 50%, 70%, and 96%). The ethanol was replaced with hexamethyldisilazane and the samples were allowed to dry overnight at 40°C. The samples from different protocols were mounted on SEM stubs, platinum-sputtered and inspected in high vacuum SEM (Jeol JSM-7600F) with a field emission gun at low voltages, to observe the structure of the cells. An EDS silicon drift detector (SDD) from Oxford (S Max, 20 mm^2^) was used and a higher voltage was applied to excite Kα.

### 2.5 Time-lapse confocal microscopy (TLCM)

For TLCM, 4 µL of the coated cells whose final OD_600_ was ∼0.4 were distributed on nutrient agar placed in a previously described specially designed chamber (de Jong et al., 2011; Rybkin et al., 2019). The chamber containing the cells was equilibrated at 37°C for 15 min and the cells were transferred to a Leica TCS SP8X confocal laser scanning microscope equipped with temperature control system cube and box that was also equilibrated at 37°C. The TLCM was performed at 1000 x magnification using an objective lens (HCX PL APO ×100/1.44 OIL) immersed in immersion oil. We monitored growth and division of each individual cell either coated or not coated (sample and control cells), that were present within the field of view using excitation at 489, and emissions at 525/25 and 750,842/33 with 850–900 V and 750–800 V gains for emGFP and Cy7 fluorescence, respectively. We measured growth rate as the function of the surface coverage by cells in time. The monitoring of individual cells using microscopy was used to distinguish either cells are dead, where they would not grow and divide at all, or they grow but with slower rates. This cannot be determined merely by the optical density measurement, since the scattered light of death encapsulated cells offset the OD values. Therefore, this results in delayed detection of changes of OD values, false longer lag phase, since only the fraction of cells that survived the treatment can scatter light after they overgrow the dead-encapsulated cells.

### 2.6 Analysis of the TLCM micrographs

To determine the growth properties of the entrapped cells in PE layers, PE toxicity and stability of the PE capsules, we analyzed their images with Fiji software (Schindelin et al., 2012). From the obtained fluorescent images forming a hyperstack of coated cells, we deleted the images that were out of the focus. The remaining hyperstacks of the images were then converted to a stack of binary images using the “make binary” plugin. On these prepared images the surface area of cells was measured at the consecutive time points by using the “analyze particles” plugin to determine temporal changes in the cell biomass. The experiments were performed in triplicate and the Student’s t-test was used for statistical comparison of the amount of cell biomass per time per group in cells with different numbers of PE layers.

### 2.7 *In vivo* biodistribution

All experiments were performed according to the relevant institutional (National Research Ogarev Mordovia State University, Russia) and international regulations of the Geneva Convention of 1985 (International Guiding Principles for Biomedical Research Involving Animals). Animal ethics clearance was approved by the decision of the ethical committee (protocol No 50 from 29.05.2017). The biodistribution of the coated bacterial cells was investigated in two groups of 3 BALB/c female mice 6–8 weeks old with the weight distribution of 20–25 g. For general anesthesia, a mixture of Zoletil (60 mg kg^−1^, Virbac SA, Carros, France) and Rometar (and 10 mg kg^−1^, Spofa, Czech Republic) was injected intraperitoneally. The intra-arterial injections of suspensions of bacteria were performed on immobilized anesthetized mice through a polyethylene catheter (PE-10 tip, Scientific Commodities INC. Lake Havasu City, Arizona), which was introduced into the right femoral artery. During the implantation the catheter was filled with isotonic sodium chloride solution. The bacterial cells were resuspended in sodium chloride solution giving a solution with OD_600_ ∼1, measured in a 200 µL well of a 96-well microtiter plate. 200µL of the bacterial suspension was administered in the mouse.

For all animals, the bacterial cells were injected in the right femoral artery and collected in the left paw by placing the permanent magnet NdFeB (1400 mT remnant magnetization, diameter–50 mm, height–20 mm) for 60 min, which was subsequently removed. The control group of animals was treated in the same way but no permanent magnet was used.

### 2.8 *In vivo* fluorescence imaging

We used an IVIS^®^ Lumina imaging system (Xenogen Corp.) to measure the distribution of Cy7 labeled bacteria with a set of filters (excitation, 710–760 nm; emission, 810–875 nm). All the fluorescence images were acquired with 0.2 s exposure. The intensities of fluorescence signals in the obtained pictures were also analyzed in Fiji software (Schindelin et al., 2012). All images were converted to a gray-scale signal using the “RGB to luminance” plugin. Prior to analysis, all the signals were normalized to the initial base level. The gray-scale signals from the paw were normalized to the signals from the abdominal area and intensities were measured over this area at a resolution of 400 pxl. Intensities of organs were measured for their entire area and normalized to the weight of the organ. The tested organs were lungs, liver, paw, kidneys, spleen, and heart. Since no signals were detected in the heart, it was not analyzed (see SM, Supplementary Figure S2). The absolute intensity of the signal from every mouse was measured according to the same procedure, except the rectangular frame was used to calculate the change in fluorescence. All experiments were performed using 3 animals per tested group.

### 2.9 Statistical analysis

All experiments were performed in triplicates. For the statistical comparison between the populations of the encapsulated cells with different numbers of PE layers the Student’s t-test was used. To determine whether data is distributed normally in experiments with mice, the normality test was applied. The obtained data of bacterial distribution within living mice and organs were analyzed by non-parametric Mann Whitney two-tailed and Kruskal–Wallis tests. The Mann Whitney test was used to compare the difference between two groups in (*i*) the signals obtained from different parts of the mice body, (*ii*) the groups of the same organs, (*iii*) groups of different organs. The Kruskal–Wallis test was applied to determine the difference between groups of organs.

## 3 Results

The surface of the bacterial cells was functionalized by electrostatic attachment of PEs labeled with fluorescent dye and magnetic NPs (Figures 1A,B). Uncoated cells were free of any NPs, but after the coating, the negatively charged magnetite NPs adsorbed on the polycation-treated bacteria provide a dense layer on the entire cell surface. To confirm the presence of paramagnetic NPs on the surface of the cells, we applied a combination of SEM (Figures 1C,D) and energy-dispersive X-ray spectroscopy (EDS) (Supplementary Materials—SM—Supplementary Table S1). In the preparation of samples of bacteria coated with PE and magnetic NPs for scanning electron microscopy (SEM), air drying was found to be one of the most convenient and relevant approaches, because the chemical fixatives interfere with the structure of the shells, causing many problems such as aggregation or release of the cellular components (data not shown). We showed by time-lapse confocal microscopy (TLCM), that the entrapped cells remain alive inside the formed PE layers. The most appropriate PE coated bacteria were those covered with 4 PEI layers (PEI^+^/PSS^−^/PEI^+^/magnetite-NPs^-^/(PEI^+^-Cy7/PSS^−^)_2_) and were then used in the injection experiment. This produces the highest possible fluorescence level, slower division rates of the cells and at the same time, keeps the aggregation of the cells during the LbL procedure as low as possible. After incubation *in vitro*, cell growth eventually led to release of bacteria from the capsule by breaking the capsule integrity at the edges, leaving fragments from PE capsules surrounding the cells (Figure 1F). On the level of a single cell, we determined that the growth rate was proportional to the number of the PE layers (Figure 1E) deposited on the bacterial surface. The slowest growth of the cells entrapped in 8 PE layers was 1.25 times less than of the uncoated cells. No toxicity was observed, but the coated cells all demonstrated 1.4 times longer lag phase on average than uncoated cells. Under the optimal conditions, the cells coated in 8 PE layers were shown to be released from the capsules only after 50 min of incubation.

**FIGURE 2:**
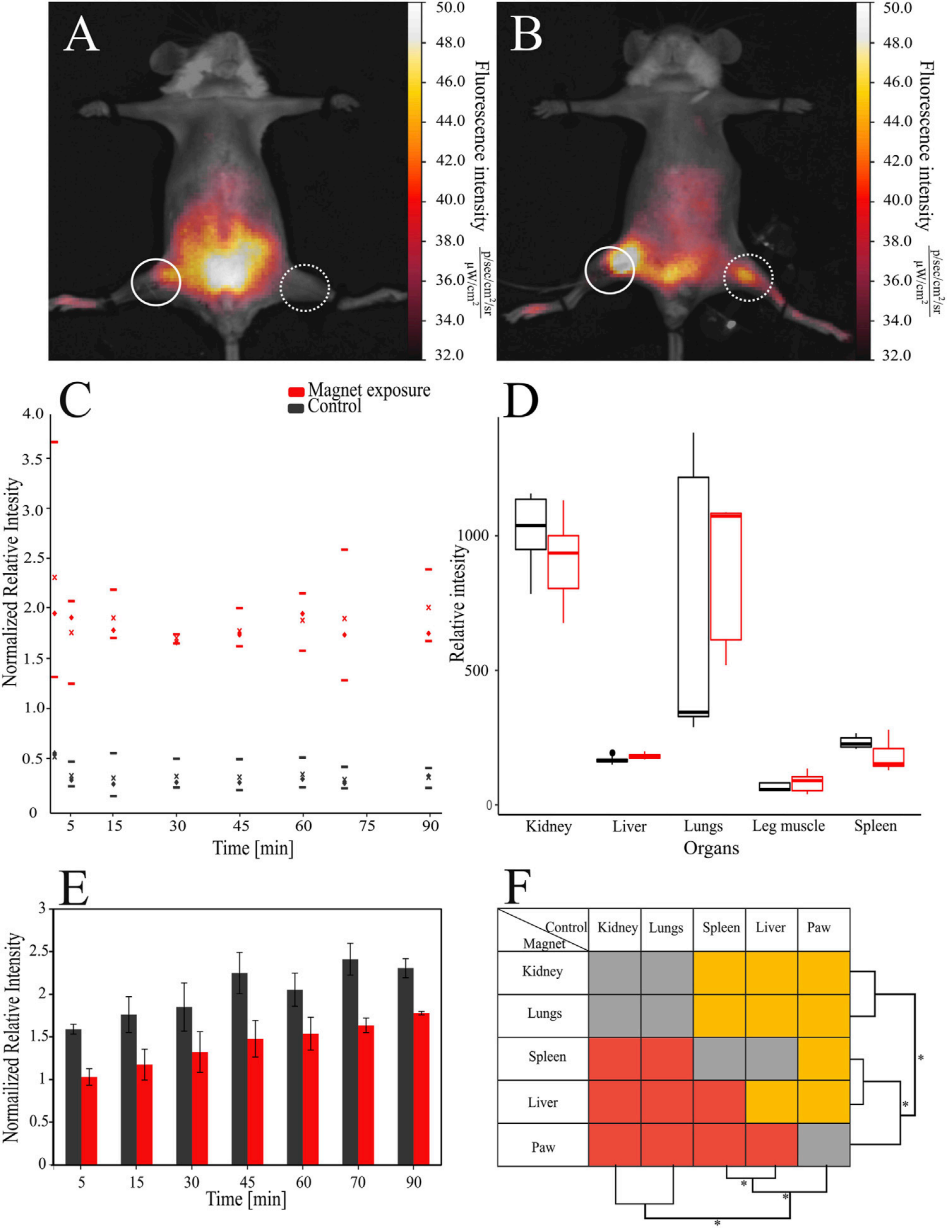
Distribution of the bacterial cells modified with magnetite nanoparticles and Cy-7 labeled PEI in mice. PEI = polyethylenimine. The bacteria were detected: **(A)** in control mice and **(B)** in exposed mice under the magnetic field, respectively, dashed circles indicate injection site, solid circles the place of cell collection. **(C)** The relative fluorescence intensity of the signal in the paw of mice exposed to the magnetic field and control mice by blood flow was used to compare the accumulation of cells, where - median, x - average, upper and lower borders are 25–75th percentile respectively. **(D)** Normalized relative fluorescence was also assessed in the organs of animals. **(E)** Introduction of bacteria led to increase of relative fluorescence intensities in murine bodies over time. **(F)** Comparison of relative intensities between different organs, red - signal of the magnet group is lower than control, gray—insignificant, orange—higher.

After administration of electrostatically functionalized bacterial cells (EFBC) into the bloodstream of mice, we observed differences in the distribution of bacteria that were dependent on the presence of the magnetic field. Upon exposure to the magnetic field, spreading of the cells in the organism from the injection site was directed toward the paw with a consequent accumulation of the bacterial cells, forming bright areas in the paw where permanent magnet was placed (Figure 2A). In other parts of the body the fluorescence signal was 2.4 times on average weaker than in the paw. In control groups, the EFBC were mainly distributed in the abdomen forming dense, bright areas, but in the intact paw with no magnetic field, the fluorescence signal was 1.8 times weaker than in other parts of the body (Figure 2B). During the magnet entrapment we were able to significantly increase the signal intensity more than 5 times in the paw region than in the control group (*p* < 0.001) (Figure 2C). Because the magnet attracted bacterial cells, we determined that an excess of up to 4.6 ± 0.1% of cells were accumulated in the paw, while the overall distribution of free circulating cells in both the control and test groups remained the same. After the magnet was removed, the signal from the exposed paw was 5.8 times higher than in the unexposed paw and remained so for at least 30 min (*p* < 0.001).

The fluorescence signal recorded from other parts of the body increased over time (Figure 2E). During the entire experiment signal intensities associated with the body in the control group were 1.4 times higher on average than those in the magnet group (see also SM, Supplementary Table S2). At the end of the experiment after 90 min, which was 30 min after the magnet was removed, the signal intensity from the rest of the body in the group with the magnetic field was 1.8 times lower than in the control setup (*p* < 0.001).

After dissection of the animals, the fluorescent signals from the vital organs were analyzed, and were found to be 1.1 times higher just in the liver in the group exposed to the magnetic field than in the control group (*p* < 0.05) (Figure 2D). The results of paired statistical comparison between organs showed that the most cells were accumulated in kidney and lungs roughly equally with the reduction of the signal in the order of liver > spleen > paw (*p* < 0.05 in inter pairs, Figure 2F) (see also *Ex-vivo* imaging of dislodged organs SM, Supplementary Figure S2).

## 4 Discussion

For the first time it has been shown that a method of surface modification of bacterial cells, by electrostatically controlled deposition of magnetite-containing PEs can increase by a factor of more than five the specificity of delivery of EFBCs to the particular site with magnetic field guidance (see Figure 2C). Comparing with similarly sized microparticles (see Mayorova et al., 2020), here the specificity was more than doubled (Mayorova et al., 2020). The difference in the specificity could be attributed to the magnetic field which was 4 times stronger than those used with similarly sized microparticles and reported previously (Mayorova et al., 2020). However, merely the attraction of bacteria to the particular place within the body does not guarantee the specific interaction of bacterial cells with specific malignant cells, although with a longer exposure time this probability should be increased (Wagner et al., 2003). In order to increase the specific interaction polyelectrolytes deposited in layers on bacterial cell walls can represent a platform to which different enzymes, receptors or antibodies can be efficiently attached or embedded within layers (Ai et al., 2003; Murelli et al., 2009; Scott et al., 2012). Such attachment is assisted in the longer interaction times induced by the magnetic field, and can result in increased specific interaction with cells expressing particular epitopes on their surface. Moreover, although encapsulated bacterial cells cannot use flagella to propel themselves toward tumors, the facilitation of EPR effect by addition of e.g., nitroglycerin in combination with the externally applied magnetic field can increase the number of bacterial cells in tumor tissue and increase efficiency of delivery (Fang et al., 2016).

Although we prepared magnetized bacterial cells where the EDS measurement showed presence of relative values of Fe signal (see Figures 1C, D; Supplementary Table S1), we were not able to determine exact amount of a dsorbed magnetite NP or amount of Fe between the PE layers. This is important to be determined before conducting any clinical studies since the disintegration of the magnetic coating, the presence of free NPs as well as PE, and the interaction of NPs with the PE as well as immune system can occur when longer exposure times are considered.

Since it was necessary to obtain a sufficient signal from injected cells *in vivo*, a high (and unrealistic) dose (8 × 10^8^ CFU) of the EFBC was injected. With the magnetic field, a relatively small fraction, 4.6% of cells were attracted and retained in the paw (see Figure 2B). The rest of the signal was distributed through the body where high values were observed in organs with big blood volumes such as kidney, lungs, liver and spleen (compare signals of organs to the heart in the Supplementary Fig. S2). This comparison of signals from those organs showed that the remaining cells were not retained in the paw but accumulated in different organs (see Figure 2D). This can be attributed to the property of the paw circulatory system, since it has a limited capacity and use of a stronger magnetic field would not be expected to significantly improve the level of bacteria within the paw and might block the local circulation causing local ischemia or even infarction. We estimate that in order to decrease the amount of free circulating bacteria we can use 1000 times fewer bacterial cells (Toso et al., 2002; Ali et al., 2018).

During the experiment at different time points we analyzed the overall body intensities of the fluorescent signals (Figure 2E), which unexpectedly, gradually increased during the experiment in both, control and experimental group, where the control group showed higher values of modes as well as more scattered data than the group where magnet was used (see values and number of modes, respectively, in Supplementary Table S2). This phenomenon of signal increase, especially observed after 70 min (see Figure 2E), could occur for at least two reasons: (*i*) rupture of the shell integrity caused either by the bacterial cell division, or interaction of shell with phagocytes, where labeled PEs can consequently get released, or (*ii*) unequal distribution of bacterial cells between different tissues, leading to saturation of the skin capillaries which are closer to the detector. Attribution to the bacterial cell division (*i*) can be excluded since the increased intensity of the fluorescence signal from groups exposed to the magnetic field was observed after 15 min. In the control groups the same increase was observed only after 30 min (*p* < 0.01). The division of the cells used in this experiment can occur no sooner than after 50 min in the most optimal conditions, which is not the case in the *in vivo* environment. If the cells were captured by the macrophages, killing 40%–70% of biomass (depending on cell type and expression profile) would require at least 2 h with subsequent decomposition for additional 6 h (Frankenberg et al., 2008).

We attributed the main reason for the gradual increase of the fluorescent signal as (ii), the slower retention of saturation of the capillaries by the group exposed to the magnetic field compared to the control group. It is known that in mice, the total surface area of capillaries (2000–27000 mm^2^) is much greater than the surface arterial vasculature (70–160 mm^2^) (Rennie et al., 2007). The blood volume in capillaries (5%) is much smaller in comparison to that in arteries (15%) and veins (64%), respectively and the blood flow rates are not high (Brown et al., 1997). We posit that in the long term, the number of freely circulating bacteria will be reduced as all the capillaries and organs are filled, depleting blood from arteries and veins. Therefore, the signal from the skin surface should be increased as coated cells are captured in capillaries. Since the capillaries are highly branched, a small difference in the concentration of free circulating cells, could eventually cause a slower saturation of the capillaries, until the magnetic field is removed, releasing attracted biomass. As the experiment was not prolonged, some cells could be still retained by the magnetic field, resulting in the lower overall intensity. In addition, since the increase of the signal intensity in the control group between mice was varied (see values of modes in Supplementary Table S2), it needs further investigation of the factors affecting the accumulation of bacterial cells in the capillaries.

At the end of the experiment, the cells in question were mainly distributed in kidney and lungs, as reported for uncoated bacteria (Clairmont et al., 2000) or particles about 1 micron in size (Decuzzi et al., 2010), or for nanosized particles, in the spleen, lungs, liver and kidney (Owens and Peppas, 2006; Balasubramanian et al., 2010; Morishita et al., 2015). Since the vascular volumes of the organs as well as blood flow are the highest in lung and kidney (Boswell et al., 2014), this could explain the observation that bacteria were mostly accumulated in these organs with subsequent reduction in liver, spleen and paw. Consequently, preferential targeting cells without any systemic dissemination will require consideration of vasculature capacity, blood flow and cell size.

Although in our experiments we modified the PE capsules to a certain extent in order to test remote guiding of bacterial cells, the approach described here can be further tested and improved. The concerns that are needed to be taken into account are haemocompatibility together with the immune response, PE toxicity against different bacterial cells and using magnetic field to direct bacteria toward deeper tissues. An important feature of bacterial therapy is concealing bacteria from the immune system in order to reduce acute toxicity and the possibility of sepsis. We envision several mechanisms that would allow the gradual engagement of the immune system: (*i*) the deposition of the PE capsules to hide the bacterial membrane and lipopolysaccharides (LPS) from direct exposure, (*ii*) adjustment of capsule thickness to control the speed of release of the cells and (*iii*) deposition of specific coatings to enhance the stealth circulation. The cell wall of bacteria is composed of LPS, muramyl dipeptides and lipoteichoic acid, which are known to be toxic in the immune system (Schletter et al., 1995). Administration of 10^9^ CFU of the native bacteria to BALB/c mice can induce 50% lethality (Ali et al., 2018). This was not observed in our experiments in which no lethal cases were observed at a similarly high dosage (8 × 10^8^ CFU) at least during the time of the experiments. We speculate that this can result from the presence of the PE shells, which could suppress the mouse immune response by protecting the LPS surface from direct contact with the immune cells. However, additional long term studies should be performed *in vivo* as well as *in vitro* to determine the efficiency of preventing the release of toxins from bacterial cells using different PEs and methods of formation of LbL shells.

Potential systemic immune response can be prevented if the portion of the released EFBCs that comes into direct contact with the immune system is controlled. Based on our *in vitro* experiments (see Figures 1E,F), it can be seen that the bacteria stay alive after being coated and their subsequent growth rates and release are proportional to the capsule thickness. Consequently, introduction of bacteria with coatings of different thickness would enable gradual release from the shell and modulation of the intensity of the response. The time of circulation of the coated cells could be prolonged even more by using specific coatings such as polyethylene glycol (PEG) or polysaccharides that reduce hydrophobicity and surface charge, which are the primary initiators of opsonization (Yoo et al., 2010). It is expected that the cell growth will occur mostly in tumor regions, where there are the most favorable growth conditions due to suppression of immunity (Clairmont et al., 2000; Barbé et al., 2005). Introduction of genetic elements for the expression of cytokines (Barbé et al., 2005), cytotoxic agents (Jiang et al., 2010) or facilitation of the detection by production of fluorescent dyes can make bacteria even more potent against tumors (Nguyen et al., 2010).

PEI used in this study showed no toxic effects (see Figure 1E and Rybkin et al., 2019; van Tatenhove-Pel et al., 2021), but it might be toxic if other therapeutically relevant bacteria are used. However, there have been demonstrated approaches to change or modify toxic PEI (see Deev et al., 2021), which indicate that the toxicity must be determined in beforehand the LbL is applied for the selected strains.

There are also needed further studies to direct bacterial cells toward the deeper tissue since we used permanent magnet that brought bacteria superficially toward the strongest magnetic field that is closer to the magnet surface. There are several options to focus magnetic field in deeper tissues using coil based electromagnetic fields, which must be further studied since focused magnetic fields might not be extremely high (Cubero et al., 2020).

Although there are needed many further tests in order to bring this approach toward the real theranostic system, this approach is also not limited to mitigation of cancer development because it allows us to use the coated bacteria as adjuvant systems of vaccines. Accordingly, if parts of the bacterial cell envelopes can enhance the immune response (Schletter et al., 1995; Acevedo et al., 2014), a higher elevation of the immunity can be expected when the whole bacterium is presented as a coated capsule while avoiding exaggeration of the immune activity which could engage the septic shock. In addition, the use of coatings can increase adherence of the probiotic bacteria (Anselmo et al., 2016) and their adjuvant activity (Wang et al., 2016; Mojgani et al., 2020).

## 5 Conclusion

We have described means of improving bacterial therapy by the electrostatic deposition of PEs on the cell surfaces. We prepared magnetically responsive bacterial cells, which are detectable in the far red spectrum. The use of a permanent magnet allowed us to increase the specificity of targeting by a factor of >5 compared to a control group. It was found that introduction of a high dose of coated cells did not cause animal death throughout the whole experiment. As the PE layers were deposited on the cells, it resulted in a controlled time of the cell division, which might be used for the gradual introduction of the coated bacteria for immunity. Such an approach of cell coating opens new possibilities for the use and control of various microorganisms and entertains new concepts in drug delivery as well as in preparation of adjuvants in vaccines.

## Data Availability

The original contributions presented in the study are included in the article/Supplementary Material, further inquiries can be directed to the corresponding author.
