# Supplementary material for: Modification of bacterial cells for in vivo remotely guided systems
Source: Front Bioeng Biotechnol. 2023 Jan 4;10:1070851. doi: 10.3389/fbioe.2022.1070851 (PMC9845715; doi:10.3389/fbioe.2022.1070851)
Supplement: Supplementary file 1 [file Table1.docx]

Graphical abstract


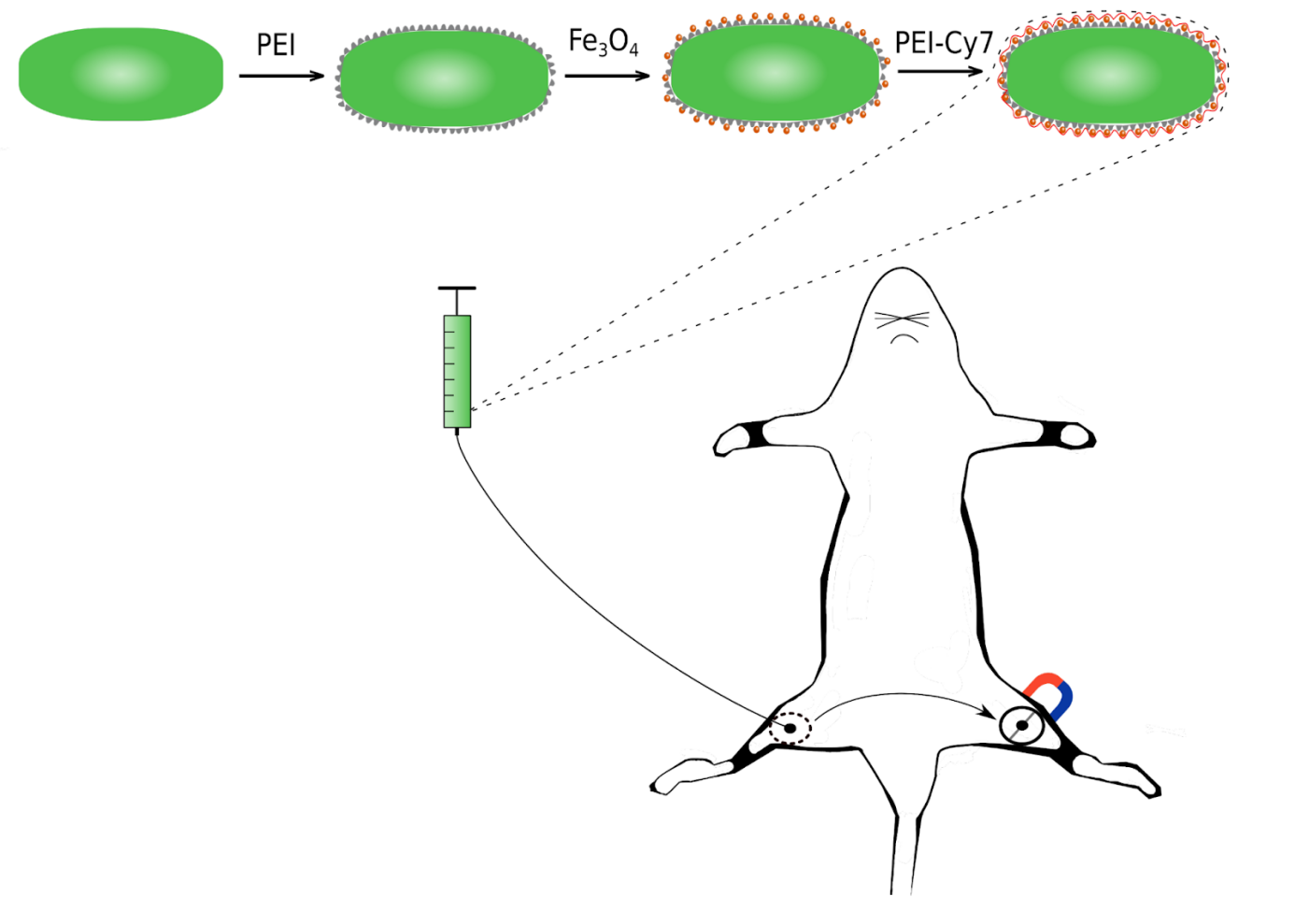


**Supplementary Table S1.**  Energy-dispersive X-ray spectroscopy **(**EDS) of bacterial cells uncoated or coated with magnetite (N/A is showing not detected elements, Si signals are from the support material and Pt from sputtering).

| Spectrum | C % | O % | Na % | Si % | Fe % | Cl % | Pt % |
| --- | --- | --- | --- | --- | --- | --- | --- |
| Spectrum 1 control | 36 | 2.9 | N/A | 60.8 | N/A | 0.1 | 0.2 |
| Spectrum 2 control | 51 | 4.7 | 0.1 | 44.1 | N/A | N/A | 0.2 |
| Spectrum 3 control | 45.1 | 4.3 | 0.1 | 50.3 | N/A | 0.1 | N/A |
| Spectrum 4 control | 35.6 | 2.8 | N/A | 61.4 | N/A | N/A | 0.2 |
| Spectrum 5 control | 37.3 | 2.7 | 0.1 | 59.6 | N/A | N/A | 0.2 |
| Spectrum 1 coated cells | 41.3 | 9 | 1 | 47.6 | 0.6 | 0.3 | 0.2 |
| Spectrum 2 coated cells | 44.6 | 10.3 | 0.6 | 43.9 | 0.3 | 0.3 | 0.1 |
| Spectrum 3 coated cells | 41.3 | 7.7 | 0.4 | 50.1 | 0.1 | 0.3 | 0.2 |
| Spectrum 4 coated cells | 40.6 | 6 | 0.3 | 52.6 | 0.1 | 0.2 | 0.2 |
| Spectrum 5 coated cells | 54.3 | 12.9 | 1 | 31.2 | 0.2 | 0.3 | 0.2 |

**Supplementary Table S2.**  Modes of relative increased intensities of 9 values taken randomly at 9 different points from the 3 mice bodies when fluorescently labeled bacteria are introduced into the murine body. NA is showing when there were not observed additional modes among the ranked data.

| Samples | Time [min] | Mode 1 | Mode 2 | Mode 3 |
| --- | --- | --- | --- | --- |
| Control mice | 5 | 1.5 | NA | NA |
|  | 15 | 2.1 | 1.8 | 1.3 |
|  | 30 | 2.2 | 2.1 | 1.2 |
|  | 45 | 2.5 | 1.7 | NA |
|  | 60 | 1.7 | NA | NA |
|  | 70 | 2.7 | NA | NA |
|  | 90 | 2.5 | 2.2 | NA |
| Mice exposed to magnet | 5 | 1 | NA | NA |
|  | 15 | 1.1 | 1.4 | NA |
|  | 30 | 1.2 | NA | NA |
|  | 45 | 1.4 | NA | NA |
|  | 60 | 1.4 | NA | NA |
|  | 70 | 1.7 | 1.6 | NA |
|  | 90 | 1.8 | NA | NA |


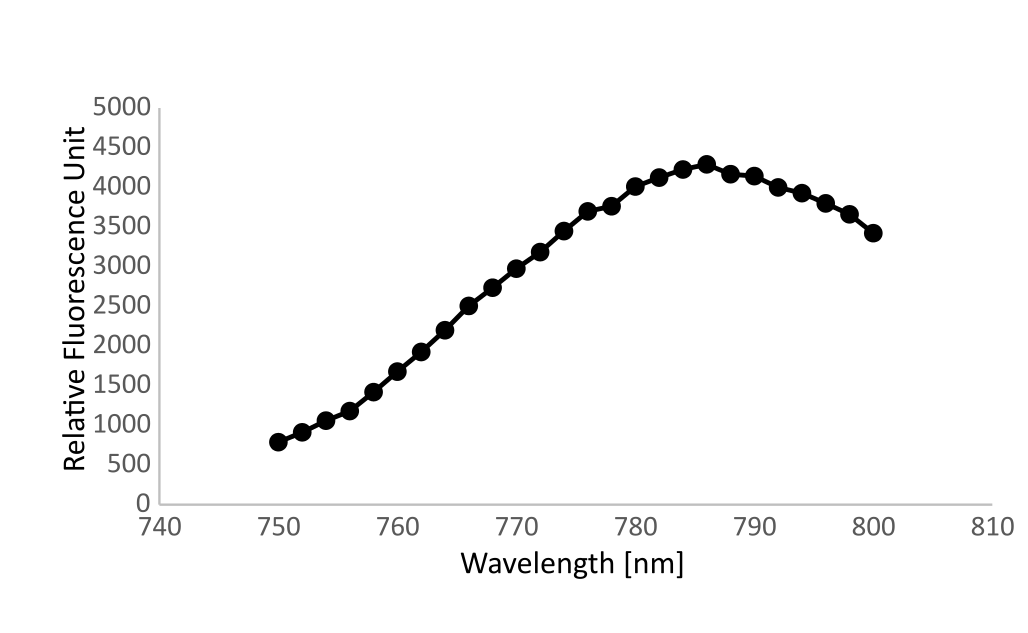


**Supplementary Figure S1**. The emission spectrum of the Polyethylenimine (PEI)-Cy7 conjugate deposited on the surface of the bacterial cells. The composition of the shell deposited on the bacterial surface was PEI/ Poly(sodium 4-styrenesulfonate) (PSS)/PEI/magnetite-NPs/(PEI-Cy7/PSS)_2_.


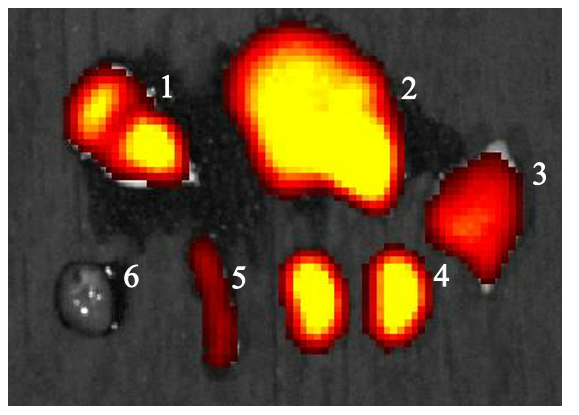


**Supplementary Figure S2**. Ex-vivo imaging of dislodged organs. 1-lungs, 2-liver, 3-paw, 4-kidneys, 5-spleen, 6-heart
